# Itaconic Acid Activates Lysosomal Biogenesis and Autophagy Flux and Mitigates High-Fat Diet-Induced Liver Lipid Accumulation in Largemouth Bass (*Micropterus salmoides*)

**DOI:** 10.3390/antiox15010006

**Published:** 2025-12-20

**Authors:** Xue Li, Shidong Wang, Muzi Zhang, Ming Li, Chao Chen

**Affiliations:** 1 Key Laboratory of Animal Genetics, Breeding and Reproduction in the Plateau Mountainous Region, Ministry of Education, Guizhou University, Guiyang 550025, China; xli8@gzu.edu.cn (X.L.); mzzhang3@gzu.edu.cn (M.Z.); 2 College of Animal Science, Guizhou University, Guiyang 550025, China; 3 College of Biosystems Engineering and Food Science, Zhejiang University, Hangzhou 310058, China; wangsd@zju.edu.cn; 4 School of Marine Sciences, Ningbo University, Ningbo 315211, China; liming1@nbu.edu.cn

**Keywords:** largemouth bass, itaconic acid, high-fat diet, lysosomal biogenesis, autophagy, fatty acids oxidation

## Abstract

This study investigated the interventional effects of dietary itaconic acid (ITA) on high-fat diet (HFD)-induced lipid deposition in largemouth bass (*Micropterus salmoides*) and the underlying mechanisms. Results showed that ITA supplementation significantly alleviated HFD-induced growth performance inhibition, as indicated by increased weight gain rate, increased specific growth rate, and reduced feed conversion ratio. ITA supplementation effectively reversed the HFD-induced increase in the hepatosomatic index, intraperitoneal fat ratio, serum triglycerides, total cholesterol, low-density lipoprotein/high-density lipoprotein ratio, hepatic lipid droplet accumulation, and hepatocyte vacuolation. Importantly, ITA ameliorated HFD-induced impairment of antioxidant capacity and reduced liver alanine aminotransferase and aspartate aminotransferase activities. Liver metabolomics revealed that ITA reduced levels of 20 fatty acids, 14 acylcarnitines, and 13 glycerides, suggesting enhanced fatty acid oxidation and reduced lipid esterification. Transcriptome sequencing and q-PCR validation demonstrated that ITA activated the AMPK/mTOR pathway, upregulating autophagy-related genes (*prkaa1*, *ulk2*, *map1lc3a*, *sqstm1*) and lysosomal biogenesis-related genes (*ap3s2*, *igf2r*, *lgmn*, *ctso*), thereby enhancing autophagic-lysosomal flux and promoting lipid degradation. In conclusion, ITA reduces hepatic lipid accumulation by synergistically activating autophagy and lysosomal biogenesis, thereby facilitating the oxidative degradation of fatty acids within lysosomes. This study provides a theoretical basis for the application of ITA as a functional feed additive in aquaculture.

## 1. Introduction

Fish fatty liver disease (FLD) refers to a range of physiological abnormalities and metabolic disturbances characterized by excessive accumulation of lipids within the liver tissue. It has become a prevalent nutritional disorder in global aquaculture, especially within Chinese aquaculture systems. FLD negatively impacts cultured fish by impairing growth metrics and feed conversion efficiency, compromising immune and stress response mechanisms, deteriorating flesh quality, and indirectly encouraging antibiotic overuse, consequently raising food safety concerns [1]. The pathogenesis of FLD is multifactorial, encompassing: nutritional and diet-related factors such as high dietary energy levels [2], deficiencies in essential nutrients [3,4], and feed oxidation processes [5]; environmental pollutants, including heavy metals [6,7] and organic contaminants [8,9], which interfere with lipid metabolic pathways; physiological variables like age and sex, which influence hepatic lipid accumulation [10,11]; interspecific variations in nutritional tolerance and lipid storage strategies [12,13]; and genetic factors, including specific gene mutations linked to fatty liver development [14]. Despite the complexity of FLD etiology, early diagnosis and targeted management strategies are crucial for mitigating its impact on aquaculture productivity.

Significant progress has been made in preventing and alleviating FLD in farmed fish. Sedum sarmentosum Bunge extract (FSSB) has been shown to markedly upregulate PPARα to promote fatty acid oxidation while downregulating PPARγ to inhibit lipid storage, thereby stimulating lipid breakdown and suppressing synthesis. Furthermore, FSSB upregulates apolipoprotein ApoA1 and fatty acid-binding proteins FABP1 and FABP3, which are involved in lipid digestion and absorption, thereby facilitating intracellular fatty acid transport and utilization. FSSB also exerts precise regulation on the expression of stearoyl-CoA desaturase and acyl-CoA-binding protein, influencing fatty acid desaturation and β-oxidation rates, which collectively promote lipid catabolism [15]. Metformin decreases hepatic lipid accumulation by inhibiting gluconeogenesis and reducing glucose-to-lipid conversion, while enhancing peripheral glucose and fatty acid utilization. It also upregulates PPARα to promote fatty acid oxidation, downregulates SREBP-1c to suppress lipogenesis, and increases apolipoprotein expression, thereby improving lipid metabolism [16]. Furthermore, Chinese herbal medicine (CHM) modulates the PPAR signaling, inducing the expression of acsl6 to promote fatty acid β-oxidation, while concurrently downregulating critical lipogenic enzymes such as FASN and ACC-2. CHM also upregulates GCK and GYS2, facilitating the diversion of glucose toward glycogen biosynthesis rather than lipogenesis. These integrated mechanisms synergistically mitigate hepatic lipid accumulation [17]. Supplementation of high-fat diets with 0.3 mg/kg selenium has been found to mitigate excessive liver lipid deposition by activating lipophagy—the autophagic degradation of lipid droplets [18]. Therefore, the modification and optimization of feed through dietary additives represent a promising strategy for ameliorating fatty liver disease in largemouth bass.

Itaconic acid (ITA) is an endogenous metabolite derived from the tricarboxylic acid (TCA) cycle, produced via the decarboxylation of cis-aconitate by aconitate decarboxylase 1, which is encoded by the immune-responsive gene 1 [19,20]. ITA has been recognized for its regulatory functions in systemic energy homeostasis and resistance to obesity [21], with growing evidence suggesting its involvement in modulating lipid metabolic pathways through multiple coordinated biochemical mechanisms. Due to its structural analogy to succinate, ITA competitively inhibits succinate dehydrogenase, thereby influencing mitochondrial metabolic flux [22]. Additionally, ITA can modify key glycolytic enzymes such as glyceraldehyde-3-phosphate dehydrogenase, aldolase A, and lactate dehydrogenase A, leading to suppressed glycolytic activity and reduced diversion of energy toward lipogenesis [23,24]. Within hepatocytes, ITA is further metabolized into itaconyl-CoA via an ATP-dependent reaction catalyzed by succinyl-CoA ligase, disrupting intracellular energy homeostasis and prompting compensatory upregulation of fatty acid β-oxidation [25]. Moreover, ITA supports mitochondrial fatty acid transport by stabilizing the CPT1a/SLC25A20/CPT2 axis; it prevents ubiquitination-mediated degradation of CPT1a, thereby maintaining sustained acylcarnitine production and ongoing β-oxidation [26]. Overall, these properties position ITA as a safe, biocompatible metabolic regulator with significant potential as a feed additive to enhance hepatic lipid metabolism and mitigate hepatic lipid accumulation.

The largemouth bass (*Micropterus salmoides*), originally native to North America, has emerged as a significant aquaculture species in China owing to its rapid growth rate, robust disease resistance, high-quality flesh, large size, and remarkable adaptability to diverse environmental conditions [27,28]. However, the widespread use of high-energy diets, coupled with the voracious feeding behavior of largemouth bass, has led to excessive lipid accumulation in the liver, emerging as one of the major challenges in its cultivation. This study systematically evaluated the therapeutic effects of ITA on FLD in largemouth bass fed a high-fat diet. The evaluation encompassed liver histological alterations, lipid deposition, liver enzyme activities, and antioxidant capacity. Additionally, transcriptomic sequencing was employed to investigate the molecular mechanisms underlying the protective role of ITA in mitigating lipid accumulation. The findings present a novel, safe intervention for FLD prevention and treatment in largemouth bass, providing essential experimental evidence and theoretical support for sustainable aquaculture development.

## 2. Materials and Methods

### 2.1. Feed Preparation

Three experimental diets were formulated using fish meal, soybean meal, and soy protein concentrate as protein sources, along with fish oil and soybean oil as lipid sources. The control diet (CON) contained 10% lipid, while the high-fat diet (HF) had a lipid content of 18%. The high-fat + itaconic acid diet (HF + ITA) also contained 18% lipid and was supplemented with 0.4% itaconic acid. The detailed feed formulations and proximate composition are presented in Table 1. The feeds were prepared as follows: all ingredients were thoroughly mixed according to the respective formulations, and then ground through an 80-mesh sieve. An appropriate amount of water was added to the mixture, which was subsequently pelleted into 2 mm diameter pellets using a laboratory pellet mill. The pellets were dried in a feed dryer and stored at −20 °C until use.

### 2.2. Experimental Fish and Sample Collection

Largemouth bass were purchased from a farm in Huzhou City, Zhejiang Province, and acclimated for two weeks in an indoor recirculating aquaculture system. At the start of the trial, 270 healthy fish of uniform size (initial body weight: 2.25 ± 0.01 g) were randomly assigned to 9 tanks, with three replicate tanks per treatment and 30 fish per tank. Fish were fed to apparent satiation twice daily at 08:30 and 16:30. Water quality was monitored twice weekly and maintained within the following ranges: temperature 27.3–29.4 °C, pH 7.70–7.83, dissolved oxygen ≥ 8.00 mg/L, and total ammonia nitrogen ≤ 0.05 mg/L. The feeding trial lasted for 8 weeks. Before sampling, fish were fasted for 24 h. The total number of fish and final individual body weight in each tank were recorded, after which fish were anesthetized with MS-222 (100 mg/L). Three fish per tank were randomly collected and stored at −20 °C for whole-body proximate composition analysis, with data averaged at the tank level. Another three fish per tank were sampled, weighed, and dissected on ice to obtain body length, body weight, liver weight, and intraperitoneal fat weight for calculating the hepatosomatic index (HSI) and lipid-to-body weight ratio; the corresponding liver samples were preserved for physiological and biochemical analyses, with all measurements averaged per tank. An additional three fish per tank were sampled, and their livers were collected and stored at −80 °C for transcriptomic, metabolomic, and gene expression analyses, all conducted on a per-tank basis. Finally, two fish per tank were randomly selected for histological assessment, and their liver and intestinal tissues were fixed in 4% paraformaldehyde for hematoxylin-eosin (H&E) and Oil Red O staining protocols. All statistical analyses were performed using tank means, with n = 3 representing the three independent tank replicates per treatment.

### 2.3. Whole Fish and Feed Proximate Composition Determination

The proximate analysis of both the whole fish and diets was conducted following the AOAC [29]. Moisture content was quantified gravimetrically through oven drying at 105 °C until a stable weight was attained. Crude protein levels were determined using the Kjeldahl method with a Kjeltec 2300 Analyzer (FOSS, Hillerød, Denmark). Crude lipid was extracted using petroleum ether in a Soxtec System HT6 (Tecator, Höganäs, Sweden). Ash content was measured by incineration in a muffle furnace at 550 °C for 12 h.

### 2.4. Analysis of Liver Biochemical Parameters and Serum Antioxidant Capacity

Liver and serum biochemical biomarkers, along with antioxidant enzyme activities, were quantified utilizing commercially available assay kits (Jiancheng Bioengineering Institute, Nanjing, China). According to the manufacturers’ protocols, the following parameters were quantified: total plasma protein (TP), serum triglycerides (TG, A110-1-1), total cholesterol (TCHO, A111-1-1), low-density and high-density lipoprotein cholesterol (LDL, HDL), alanine and aspartate aminotransferase (ALT, AST), along with key antioxidant and oxidative stress markers (total antioxidant capacity, T-AOC; glutathione peroxidase, GPx; superoxide dismutase; SOD; malondialdehyde, MDA; catalase, CAT). Absorbance measurements were acquired utilizing a PT-3502C microplate spectrophotometer (Beijing Putian New Bridge Technology Co., Ltd., Beijing, China), and analyte concentrations were determined based on the respective calibration curves supplied within the assay kits.

### 2.5. Histopathological Examination

Liver samples (n = 3 per group) were fixed in 4% formaldehyde for 24 h, then paraffin-embedded and sectioned (4 μm thickness) for hematoxylin and eosin staining. The sections were deparaffinized in xylene, rehydrated through a graded ethanol series, stained with hematoxylin for 5 min and eosin for 2 min, followed by dehydration, clearing, and mounting with neutral balsam. Separately, additional liver tissues were fixed in 4% paraformaldehyde at 4 °C overnight, followed by PBS washing, dehydration in 60% isopropanol, and Oil Red O staining for 3 h in darkness. Post-staining, samples were rinsed, dehydrated, and mounted with glycerin. Imaging was conducted with an HTC5.0 CCD camera integrated with WT1000GM imaging software, version 3.5. Oil Red O-stained droplets and vacuoles were observed under a light microscope (Leica Microsystems, Wetzlar, Germany), and their percentage area was quantified using ImageJ. Ten random fields per sample were further analyzed with ImageJ software, version 2.

### 2.6. Untargeted Metabolomics Analysis

Liver tissue samples from the CON, HF, and HF + ITA groups were collected, with three biological replicates (individual fish) randomly selected per group for analysis. The frozen samples stored at −80 °C were thawed at 4 °C. Metabolomic profiling was performed by Applied Protein Technology Co., Ltd. (Shanghai, China). The raw mass spectrometry data were converted to the mzXML format using MSConvert. Peak detection, alignment, and quantification were conducted using XCMS. Differential metabolites between comparison groups were identified using MetaboAnalyst (https://www.metaboanalyst.ca/, accessed on 21 June 2025). Specifically, a two-sided multiple *t*-test followed by Benjamini–Hochberg false discovery rate (FDR) correction was applied. Metabolites meeting the criteria of |fold change| > 1.0 and an FDR-adjusted q-value < 0.05 were considered statistically significant. Subsequently, KEGG pathway enrichment analysis was performed on these significant metabolites, and pathways with a *p* < 0.05 (from the enrichment analysis) were identified as significantly perturbed.

### 2.7. Transcriptomics Analysis

Liver tissue samples were obtained from the CON, HF, and HF + ITA groups, with three biological replicates randomly selected per group. Total RNA was extracted using Trizol reagent according to the manufacturer’s instructions, and its concentration was quantified by NanoDrop spectrophotometry (Thermo Fisher Scientific, Wilmington, Delaware, USA). The sequencing libraries and high-throughput sequencing were performed by Applied Protein Technology Co., Ltd. (Shanghai, China) following the standard Illumina workflow. Libraries were prepared using a poly(A) enrichment strategy. Briefly, mRNA was fragmented and reverse-transcribed into cDNA using random hexamer primers, followed by second-strand synthesis, AMPure XP bead purification, size selection, and PCR amplification. After passing quality control, the libraries were sequenced on an Illumina platform to generate 150 bp paired-end reads, yielding approximately 40 million raw reads per sample. To ensure reproducibility, data processing followed a standardized pipeline. Raw reads were first filtered and trimmed using FASTP (v0.23.2). Clean reads were then aligned to the largemouth bass reference genome using HISAT2 (v2.2.1) with default settings. Gene-level read counts were obtained with FeatureCounts (subread v2.0.3). Gene expression was estimated as fragments per kilobase of transcript per million mapped fragments (FPKM). Differential expression analysis was performed using DESeq2 (v1.40.2). Group comparisons were conducted using a likelihood ratio test based on a negative binomial generalized linear model, and *p*-values were adjusted by the Benjamini–Hochberg method. Genes with |log_2_ fold change| > 1 and adjusted *p*-value < 0.05 were considered significantly differentially expressed. For downstream functional analysis, largemouth bass transcripts were aligned to the zebrafish proteome using BLASTX (v2.13.0) to identify homologous genes. Based on zebrafish annotations, GO and KEGG enrichment analyses were performed using ClueGO with all expressed genes as the background set.

### 2.8. RNA Extraction and qPCR Analysis

Upon confirmation of RNA quality by NanoDrop and gel electrophoresis, cDNA was synthesized (TransScript^®^ kit, TransGen Biotech Co., Ltd., Beijing, Beijing, China). The qPCR was carried out on an Applied Biosystems StepOnePlus instrument (Thermo Fisher Scientific, Foster City, California, USA) with Green qPCR Master Mix in a 20 μL volume. The protocol included a 60 s hot start at 95 °C and 40 cycles of denaturation (95 °C, 10 s) combined with annealing/extension (60 °C, 30 s). Gene expression was normalized to β-actin and EF1α and quantified using the 2^−ΔΔCt^ method [30]. All primers (Table 2) were supplied by Sangon Biotech (Shanghai, China).

### 2.9. Statistical Analysis

Statistical analysis and graphing were performed using GraphPad Prism software (version 9.0.2, Franklin Street, Boston, MA, USA). For comparisons between two groups, statistical significance was assessed using Student’s *t*-test. For comparisons involving three or more groups, a one-way ANOVA was employed. Prior to ANOVA, the assumptions of normality and homogeneity of variance were verified using the Shapiro–Wilk and Levene’s tests, respectively. If the overall ANOVA was significant, post hoc Duncan’s multiple range test was applied for pairwise comparisons. A *p*-value of less than 0.05 was considered statistically significant. Each experiment included three biological replicates. Data are presented as mean ± standard deviation (mean ± SEM).

## 3. Results

### 3.1. Effects of ITA Supplementation in HFD on the Growth Performance

Compared to the CON group, the HF group exhibited significantly reduced growth metrics (final body weight, FBW; weight gain, WG; weight gain rate, WGR; specific growth rate, SGR) and increased feed conversion ratio (FCR) (*p* < 0.05). The HF + ITA group showed significant improvements in these parameters relative to HF (*p* < 0.05), with no difference in feed intake (Table 3; *p* > 0.05). Body length (BL) was lower in HF than CON (*p* < 0.05), while liver weight (LW), hepatosomatic index (HSI), condition factor (CF), abdominal fat weight (AFW), and the ratio of abdominal fat weight to body weight (ABR) were higher (*p* < 0.05). The HF + ITA group had increased BL and decreased LW, HSI, CF, AFW, and ABR compared to HF (Table 4; *p* < 0.05).

### 3.2. Effects of ITA Supplementation in HFD on Liver Physiological Indicators of Largemouth Bass

Compared with the CON, the HF showed significant increases in TP, TG, TCHO, LDL, HDL, ALT, and AST levels (*p* < 0.05). In contrast, the HF + ITA group showed significant decreases in these parameters compared to the HF group (Table 5; *p* < 0.05).

### 3.3. Effects of ITA Supplementation in HFD on Serum Antioxidant Indexes and Whole-Body Composition of Largemouth Bass

The HF group exhibited markedly elevated levels of H_2_O_2_ and MDA relative to the CON group, accompanied by a significant suppression of key antioxidant enzyme activities including T-AOC, SOD, CAT, and GPX (*p* < 0.05). Conversely, supplementation with ITA in the HF + ITA group effectively mitigated oxidative stress, as evidenced by decreased H_2_O_2_ and MDA concentrations and a concurrent restoration of antioxidant enzyme activities compared to the HF group (*p* < 0.05, Table 6). The crude lipid content in HF group was significantly higher than that in the CON group (*p* < 0.05, Table 7), while the crude lipid content in the HF + ITA group was significantly lower than that in the HF group (*p* < 0.05, Table 7).

### 3.4. Effects of ITA Supplementation in HFD on Liver Tissue Morphology of Largemouth Bass

**Figure 1:**
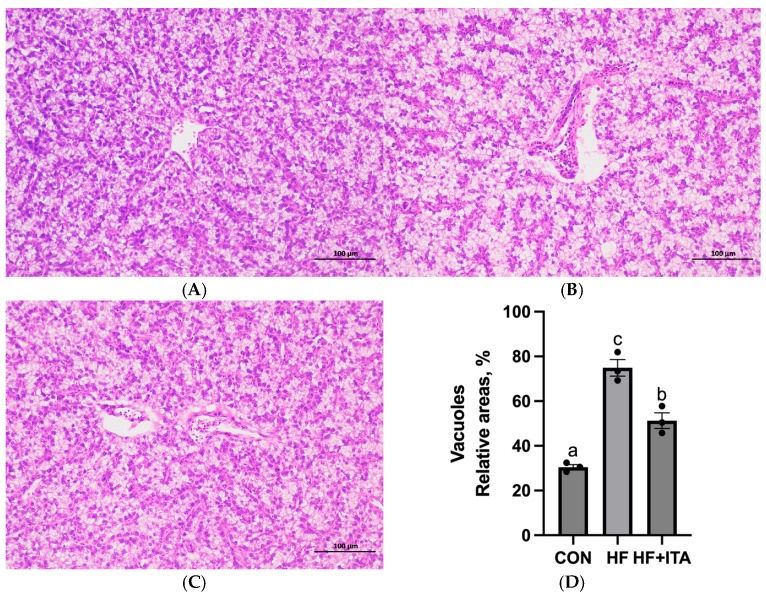

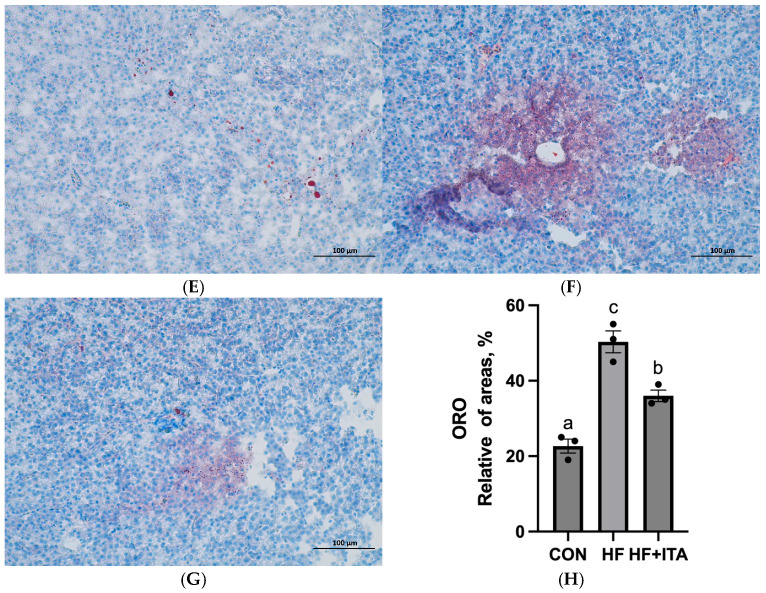
Effects of dietary supplementation with 0.4% ITA on liver histopathological sections in largemouth bass fed a high-fat diet. Representative H&E staining of liver tissue sections in (**A**) CON, (**B**) HF and (**C**) HF + ITA groups (scale bar, 100 μm). (**D**) Quantitative analysis of vacuolization area in liver sections. ORO staining of liver tissue sections in the (**E**) CON, (**F**) HF and (**G**) HF + ITA groups (scale bar, 100 μm). (**H**) Quantitative analysis of the area of lipid droplets in liver sections. Lipids appear red and nuclei appear blue after Oil Red O staining. The depth of red color and the size of the lipid droplets were positively correlated with the lipid content. Statistical differences are represented by different letters (*p* < 0.05).

Histological H&E staining revealed significant hepatocellular vacuolization progression in the HF group compared to CON (Figure 1A,B), indicating HFD-induced hepatic injury. Notably, this morphological abnormality was remarkably mitigated by ITA supplementation (Figure 1C,D). These results suggest that ITA can alleviate the structural liver damage induced by HFD. Assessment of Oil Red O-stained liver sections revealed that the area of lipid droplet accumulation (red-stained areas) was markedly increased in the HF group compared with the CON group (Figure 1E,F). In contrast, this lipid deposition was substantially reduced in the HF + ITA group relative to the HF group (Figure 1G,H). Collectively, these data indicate that ITA alleviates the pathological lipid deposition in the liver induced by a high-fat diet.

### 3.5. Effects of ITA Supplementation in HFD on Liver Lipid Metabolomics

**Figure 2:**
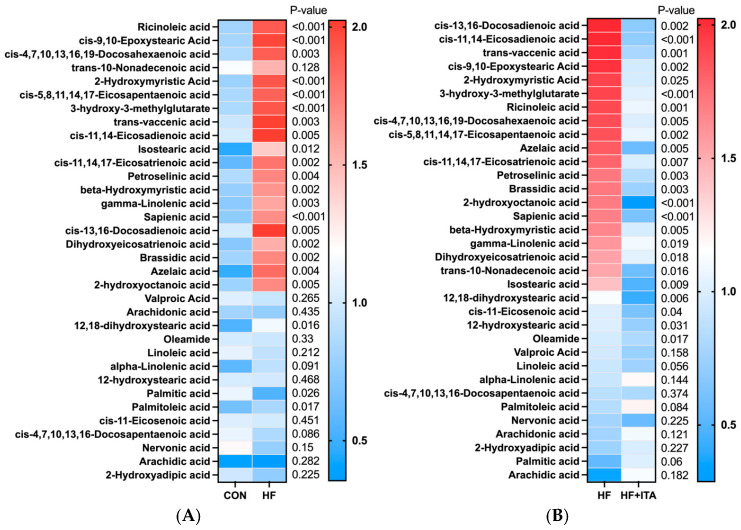
Effects of dietary supplementation with 0.4% ITA on metabolites related to lipid metabolism (fatty acids) in the liver of largemouth bass fed a high-fat diet (n = 3). Metabolomic profiling of the liver was performed on three randomly selected specimens from the CON, HF, and HF + ITA. The heat map illustrates the normalized intensities of fatty acids. Statistical significance was determined at *p* < 0.05 for comparisons between (**A**) CON vs. HF and (**B**) HF vs. HF + ITA.

**Figure 3:**
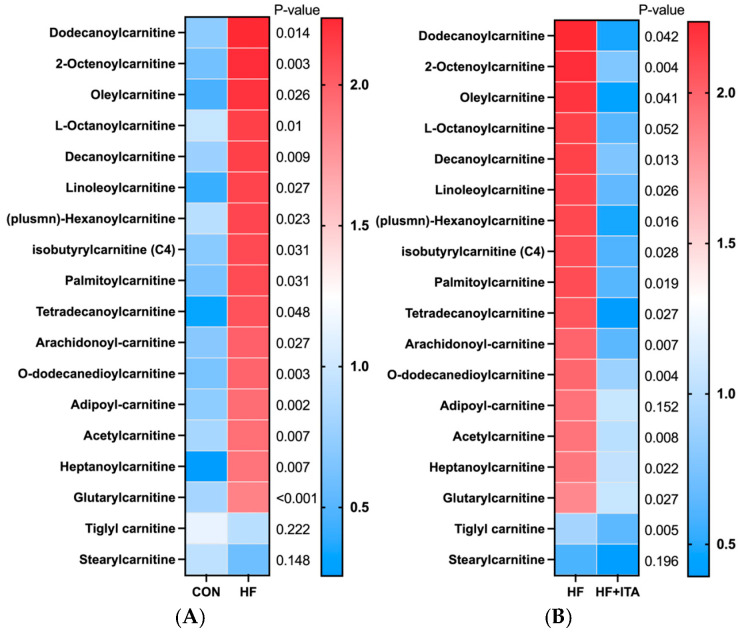

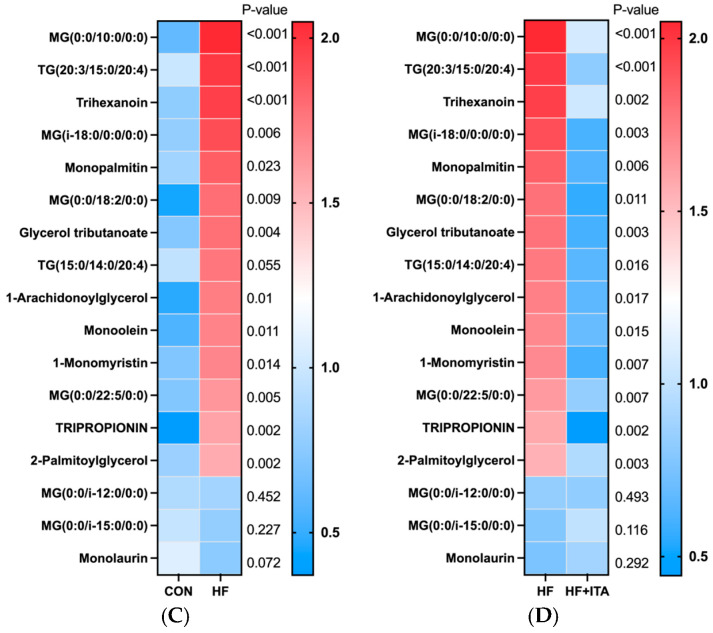
Effects of dietary supplementation with 0.4% ITA on metabolites related to lipid metabolism (acylcarnitine and glyceride) in the liver of largemouth bass fed a high-fat diet (n = 3). The relative abundance of liver acylcarnitine in (**A**) CON vs. HF and (**B**) HF vs. HF + ITA. The relative abundance of liver glyceride in (**C**) CON vs. HF and (**D**) HF vs. HF + ITA. Livers from three fish from the CON, HF, and HF + ITA groups were analyzed for metabolomics using mass spectrometry. The heat map depicts the normalized value. A significant difference in the CON vs. HF and HF vs. HF + ITA groups was considered when *p* < 0.05, as determined by two-sided multiple *t*-tests (one per row).

This study conducted a heatmap analysis on metabolites related to fatty acid metabolism in the liver. The HF group exhibited higher relative levels of 19 fatty acids (such as ricinoleic acid and cis-9,10-epoxystearic acid) than the CON group (*p* < 0.05), as summarized in Figure 2. The HF + ITA group demonstrated a marked decline in the levels of 24 fatty acids (such as cis-13,16-docosadienoic acid and cis-11,14-eicosadienoic acid) compared to the HF group (Figure 2). As shown in Figure 3, compared to the CON group, the relative abundance of 16 acylcarnitines (Dodecanoylcarnitine, 2-Octanoylcarnitine, etc.) significantly increased in the HF group (*p* < 0.05). Compared to the HF group, 14 acylcarnitines significantly decreased in the HF + ITA group (Dodecanoylcarnitine, 2-Octanoylcarnitine, etc.) (*p* < 0.05). Compared to the CON group, 13 glycerides (MG (0:0/10:0/0:0), TG (20:3/15:0/20:4), etc.) relative abundance were significantly increased in the HF group (Figure 3, *p* < 0.05). Compared to the HF group, 14 glycerides significantly decreased in the HF + ITA group (MG (0:0/10:0/0:0), TG (20:3/15:0/20:4), etc.) (Figure 3, *p* < 0.05).

### 3.6. Effects of ITA Supplementation in HFD on Liver Transcriptomics

**Figure 4:**
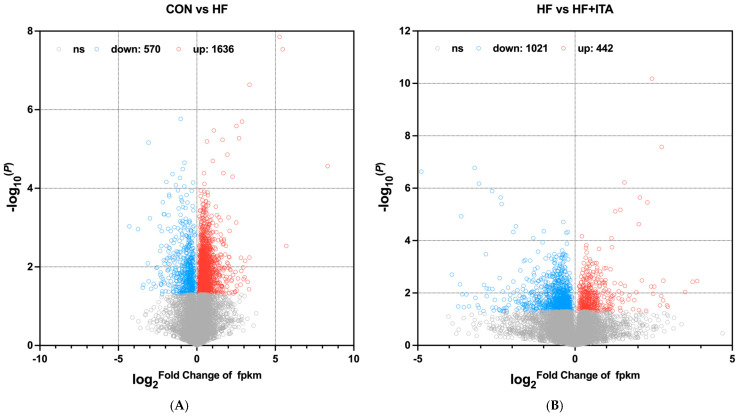

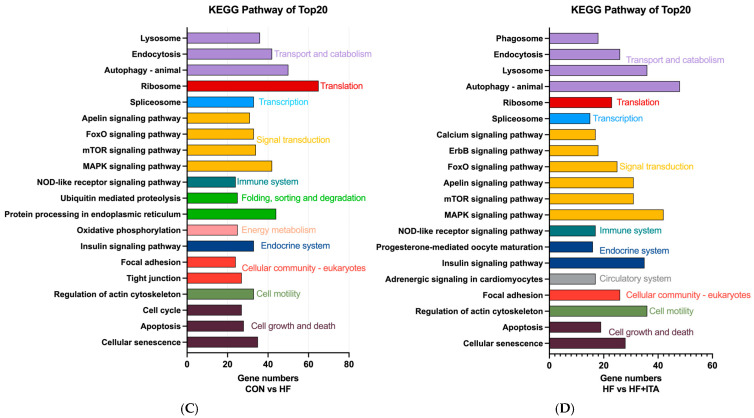
Effects of dietary supplementation with 0.4% ITA on liver gene transcription in largemouth bass fed a high-fat diet. Volcano map of differentially expressed genes (DEGs) between (**A**) CON vs. HF and (**B**) HF vs. HF + ITA groups. KEGG enrichment analysis of DEGs between (**C**) CON vs. HF and (**D**) HF vs. HF + ITA groups.

As shown in Figure 4A, a total of 2206 differentially expressed genes (DEGs) were detected in the comparison between CON vs. HF groups, including 570 significantly down-regulated genes and 1636 significantly up-regulated genes (*p* < 0.05). Analysis of the HF and HF + ITA comparison revealed 1463 differentially expressed genes, with a distribution of 1021 significantly down-regulated and 442 up-regulated genes (Figure 4B) (*p* < 0.05). Enrichment analysis of DEGs (Figure 4C,D) indicates that HF significantly modulates hepatic Transport, Catabolism, and Signal Transduction pathways. Specifically, in Transport and Catabolism, pathways such as Lysosome, Endocytosis, Autophagy-animal, and Phagosome are enriched. In Signal Transduction, pathways including mTOR and MAPK are prominently affected. The addition of ITA to HFD similarly influences these pathways, with enrichment in six Signal Transduction pathways and four in Transport and Catabolism.

### 3.7. Effects of ITA Supplementation in HFD on mTOR Signaling Pathway

**Figure 5:**
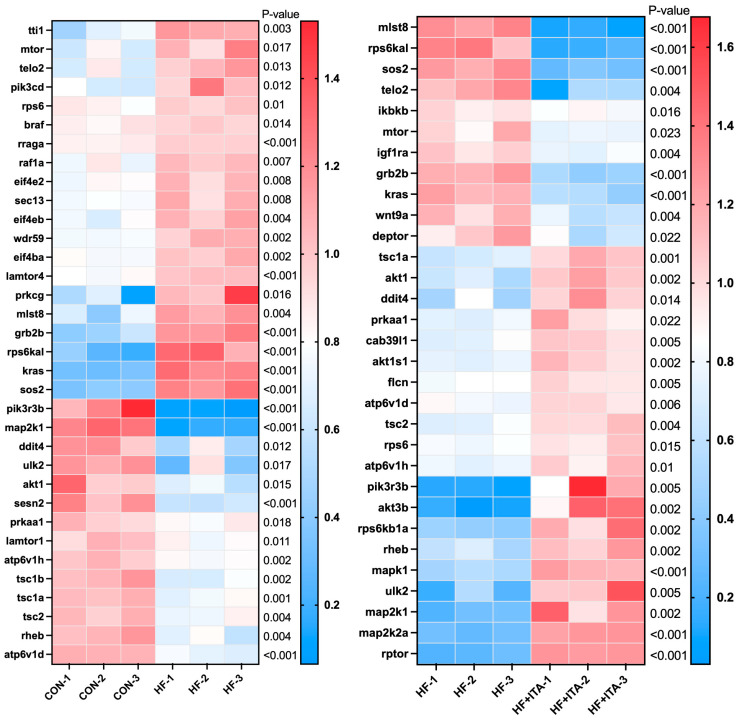
Effects of dietary supplementation with 0.4% ITA on the liver mTOR signalling pathway in largemouth bass fed a high-fat diet (n = 3). Gene expression profiles in CON vs. HF and HF vs. HF + ITA groups.

Transcriptome analysis (Figure 5) revealed that, relative to CON, the HF group exhibited significant upregulation of *mTOR*, *telo2*, *wdr59*, and *mlst8*, while *ulk2*, *tsc1b*, *tsc1a*, *tsc2*, and *rheb* were markedly downregulated (*p* < 0.05). The fragment counts of genes such as *mlst8*, *telo2*, *mTOR*, and *deptor* were significantly decreased in the HF + ITA group compared with the HF group, while the fragment counts of genes such as *tsc1a*, *tsc2*, *rheb*, *ulk2*, and *rptor* were significantly up-regulated (*p* < 0.05). HFD markedly down-regulated the expression of multiple genes in the “mTOR signaling pathway”, an effect that was significantly reversed upon ITA supplementation (Supplemental Figure S1A,B).

### 3.8. Effects of ITA Supplementation in HFD on the Autophagy-Animal

**Figure 6:**
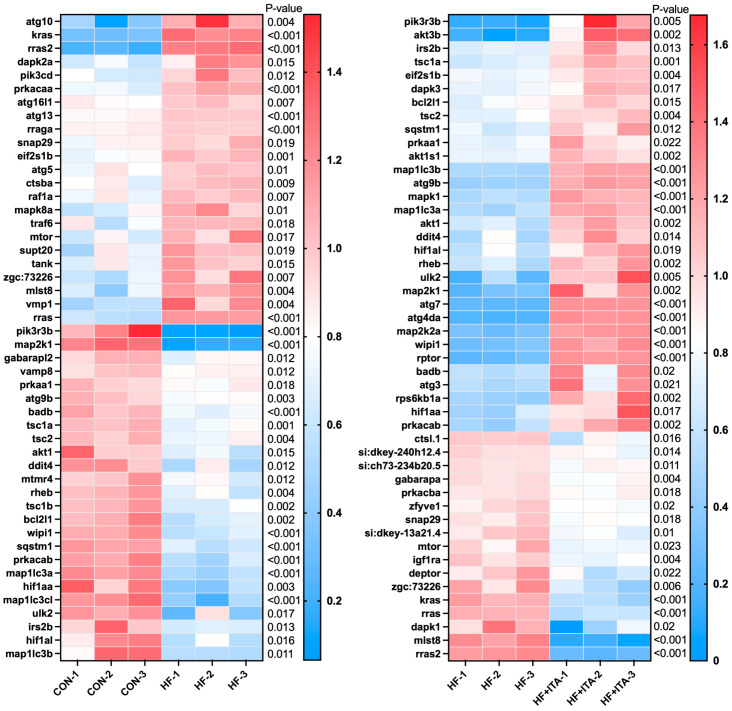
Effects of dietary supplementation with 0.4% ITA on liver autophagy genes in largemouth bass fed a high-fat diet (n = 3). Gene expression profiles in CON vs. HF and HF vs. HF + ITA groups.

We found that “Autophagy-animal” was present in both comparison pairs: CON vs. HF and HF vs. HF + ITA. Transcriptome data (Figure 6) showed that the fragment counts of *atg10*, *atg16l1*, *atg13*, *atg5*, *mTOR*, and other genes were significantly upregulated in the HF group compared to the CON group (*p* < 0.05). The fragment counts of *atg9b*, *tsc1a*, *tsc2*, *akt1*, *rheb*, *tsc1b*, *wipi1*, *sqstm1*, *map1lc3a*, *map1lc3cl*, *ulk2*, *map1lc3b* and other genes were significantly down-regulated (*p* < 0.05). The fragment counts of *tsc1a*, *tsc2*, *sqstm1*, *map1lc3b*, *atg9b*, *map1lc3a*, *rheb*, *ulk2*, *atg7*, *atg4da*, *wipi1*, *rptor*, and *atg3* were significantly upregulated in the HF + ITA group compared to the HF group (*p* < 0.05). The *mTOR*, *deptor*, and *mlst8* were significantly decreased in the HF + ITA compared to HF (*p* < 0.05) groups. HFD markedly suppressed multiple “Autophagy-animal” genes (Supplemental Figure S2A), with ITA supplementation significantly reversing these reductions (Supplemental Figure S2B).

### 3.9. Effects of ITA Supplementation in HFD on the Lysosome

**Figure 7:**
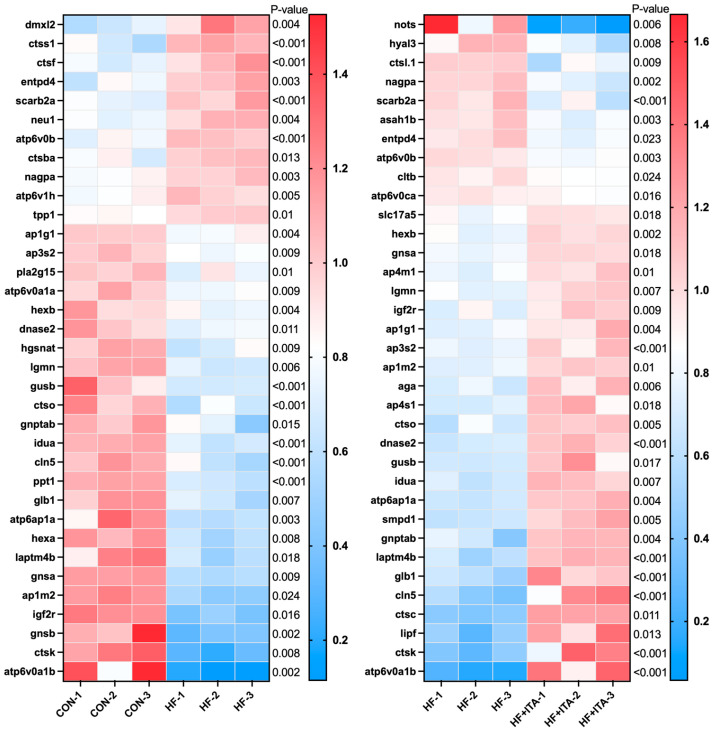
Effects of dietary supplementation with 0.4% ITA on liver lysosomes of largemouth bass fed a high-fat diet (n = 3). Gene expression profiles in CON vs. HF and HF vs. HF + ITA.

We found that “Lysosome” was present in both comparison pairs: CON vs. HF and HF vs. HF + ITA. Transcriptome data showed (Figure 7) that compared with the CON group, the fragment counts of genes *dmxl2* and *entpd4* were increased in the HF group, while *ap1g1*, *ap3s2*, *atp6v0a1a*, *lgmn*, *ctso*, *ap1m2*, *igf2r*, *gnsb*, and *ctsk* were significantly decreased (*p* < 0.05). Compared with the HF group, the fragment counts of genes such as *nots* and *nagpa* were significantly down-regulated in the HF + ITA group (*p* < 0.05), while the fragment counts of genes such as *gnsa*, *ap4m1*, *lgmn*, *igf2r*, *ap1g1*, *ap3s2*, *ap1m2*, *ap4s1*, *ctso*, *ctsc*, and *ctsk* were significantly up-regulated (*p* < 0.05). Mapping the genes contained in “Lysosome” to KEGG signaling pathways (Supplemental Figure S3A) revealed that HFD significantly reduced the expression of multiple genes in “Lysosome”. As shown in Supplemental Figure S3B, these reduced gene fragments were significantly reversed after supplementing ITA in the HFD.

### 3.10. Quantitative PCR Analysis

**Figure 8:**
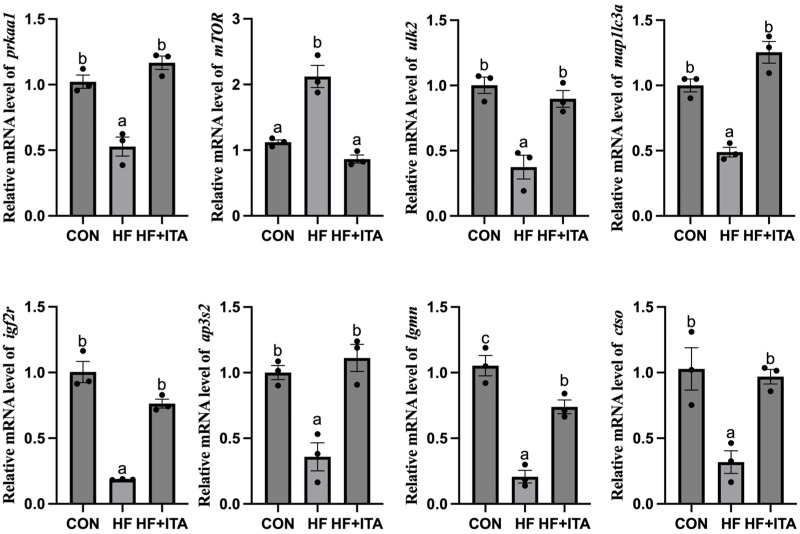
Quantitative PCR analysis of key genes involved in the AMPK/mTOR-lysosome/autophagy pathway and lysosome pathway (n = 3). Differences were deemed statistically significant at a *p*-value threshold of less than 0.05 (marked by different letters).

Coordinated downregulation was observed in the HF group for genes spanning the AMPK/mTOR-Autophagy-Lysosome axis (*prkaa1*, *ulk2*, *map1lc3a*, *igf2r*, *ap3s2*, *lgmn*, *ctso*), while mTOR expression was conversely upregulated. These expression trends were significantly reversed by dietary ITA supplementation (*p* < 0.05; Figure 8).

## 4. Discussion

FLD has historically posed a significant challenge in aquaculture [31]. Under intensive rearing conditions, declining water quality and overfeeding with high-energy diets rapidly induce hepatic lipid accumulation, impairing product quality and economic profitability while compromising immune defenses and threatening industry sustainability. Current research on ITA in aquatic species primarily focuses on its anti-inflammatory properties [32], with limited evidence regarding its role in lipid metabolism [33]. Our findings demonstrate that ITA alleviates hepatic lipid accumulation induced by high-fat diets through activation of the AMPK/mTOR signaling pathway, promoting lipophagy and upregulating genes involved in lysosome biogenesis.

Numerous studies have confirmed that long-term consumption of HFD suppresses the growth performance of largemouth bass by impairing intestinal health and mitochondrial function [34,35]. The HF group displayed significantly depressed growth metrics (WG, WGR, SGR) and a concomitant increase in FCR compared to the CON group. Collectively, these findings demonstrate that the high-fat diet markedly impaired growth and compromised feed efficiency in largemouth bass. In contrast, dietary supplementation with ITA significantly enhanced WG, WGR%, and SGR%, and reduced FCR compared to the HF group, demonstrating that ITA alleviates the impaired growth performance induced by the HFD. These findings are consistent with previous research showing that the addition of the 4-octyl itaconate significantly improved growth performance in Nile tilapia [32]. The HFD group showed significantly higher LW, HSI, CF, AFW, and ABR compared to the CON group. Furthermore, H&E staining indicated increased white vacuoles in the HFD group, and Oil Red O staining showed larger red areas, indicative of lipid droplets. These results suggest that the HFD induced abnormal lipid accumulation in the liver, which aligns with observations reported in gibel carp (*Carassius gibelio*) [36]. ITA can effectively alleviate fatty liver-related pathological conditions and mitigate associated adverse physiological effects in mammals [25,37]. In the present study, fish in the HF + ITA group exhibited significantly lower LW, HSI, CF, AFW, and ABR compared to the HF group. H&E-stained liver sections showed a reduced number of white vacuoles, and Oil Red O staining indicated a significant decrease in the red areas corresponding to lipid droplets. These findings demonstrate that ITA effectively alleviates HFD-induced lipid accumulation. Previous studies have also reported that ITA ameliorates HFD-induced obesity and lipid deposition in mammals [38].

Serum levels of TCHO, TG, ALT, and AST are key parameters for evaluating liver health [39,40] and lipid metabolism status in largemouth bass [41]. In the present study, significantly elevated levels of TG, TCHO, ALT, and AST were observed in the HF group compared to the CON group, indicating that the HFD induced both lipid accumulation and liver tissue damage in largemouth bass. In contrast, these parameters were significantly reduced in the HF + ITA group compared to the HF group, demonstrating that ITA supplementation effectively ameliorated liver steatosis and injury caused by the HFD. Our results are in line with previous work demonstrating that ITA counteracts HFD-induced increases in serum ALT/AST [42] and attenuates liver accumulation of TG and TCHO [43], reinforcing its well-established role in mitigating metabolic disturbances. LDL primarily mediates liver-to-peripheral cholesterol transport, while HDL facilitates reverse cholesterol transport, returning peripheral cholesterol to the liver for catabolism [44]. In the current study, HFD was found to significantly reduce LDL levels while increasing HDL levels in the liver, indicating a disruption in liver lipid transport functions. Supplementation with ITA reversed this trend, indicating that ITA enhances the efficiency of fatty acid transport and metabolism in the liver. Antioxidant capacity is an important indicator for assessing growth and physiological status in fish, and it directly reflects whether oxidative stress occurs during cultivation [45]. In this study, the HFD significantly increased the levels of H_2_O_2_ and MDA, while decreasing the activities of T-AOC, SOD, CAT, and GPX, indicating that the HFD induced severe oxidative stress. ITA reversed these changes, suggesting that ITA effectively alleviates the decline in antioxidant capacity caused by the HFD. Previous studies have shown that HFD can cause mitochondrial rupture and inhibit mitophagy in the liver of spotted seabass (*Lateolabrax maculatus*) [46]. The damaged mitochondria then produce excessive ROS, which further aggravates hepatocellular injury. In contrast, ITA has been reported to promote lysosomal biogenesis [47] and mitophagy [48], thereby enhancing the removal of damaged mitochondria, reducing ROS accumulation resulting from mitochondrial disruption, and mitigating subsequent cellular and mitochondrial damage. This mechanism may partially explain how ITA improves antioxidant capacity, although further studies are needed to confirm this pathway. In addition, earlier reports have also demonstrated that ITA can reduce oxidative stress induced by heat stress [49] and pathogenic bacteria [50], further supporting its beneficial role in maintaining cellular redox balance.

To further investigate the impact of ITA on liver lipid metabolism, we quantitatively analyzed the composition and content of fatty acids in the liver using metabolomics techniques. The results revealed that among the 34 fatty acids detected in both the CON and HF groups, 20 were significantly elevated in the HF group compared to the CON group, indicating that the HFD markedly suppressed fatty acid catabolism in the liver. Consistent with previous studies, HFD has been shown to significantly increase the levels of polyunsaturated fatty acids such as DHA and EPA in the liver of blunt snout bream (*Megalobrama amblycephala*) [51,52]. Acylcarnitines are vital carriers facilitating mitochondrial entry of long-chain fatty acids for β-oxidation, playing a crucial role in fatty acid metabolic pathways. Among the 18 acylcarnitines detected in this study, 14 were significantly increased in the HF group compared to the CON group. This suggests that the HFD led to a substantial influx of exogenous fatty acids into the liver. In response to this lipid overload, the liver employs both enhanced fatty acid oxidation and esterification storage pathways to maintain metabolic homeostasis [53]. The observed increase in acylcarnitine levels suggests an enhanced transport of fatty acids into the mitochondria for subsequent β-oxidation. However, the persistent and abnormal rise in acylcarnitines also reflects mitochondrial overload and a saturation of oxidative capacity, implying a potential bottleneck in the metabolic flux [54,55]. When the fatty acid oxidation pathway becomes saturated, excess lipids are redirected into esterification, promoting glyceride synthesis and accumulation in the liver, which can ultimately lead to liver steatosis [18]. Among the 17 glycerides detected, 13 showed a significant increase in the HF group compared to the CON group. This result indicates that HFD induces substantial exogenous fatty acid influx, and the liver capacity for fatty acid oxidation becomes relatively insufficient. As β-oxidation and other oxidative pathways approach saturation, surplus fatty acids are shunted into esterification, promoting glyceride synthesis. This process represents a compensatory adaptive mechanism whereby the liver stores excess fatty acids as glycerides to alleviate lipotoxicity caused by free fatty acid accumulation [56]. Notably, ITA can significantly reduce the levels of fatty acids, acylcarnitines, and glycerides in the liver fed HFD, demonstrating that ITA effectively alleviates liver lipid deposition. In line with previous findings, ITA may mitigate liver lipid burden by promoting fatty acid catabolism [25,26] and optimizing the distribution of lipid metabolic fluxes [24], suggesting its potential role in maintaining lipid metabolic homeostasis.

To elucidate the molecular mechanisms by which ITA facilitates fatty acid degradation, we conducted transcriptomic sequencing of liver tissues to examine lipid-related regulatory pathways. Compared with the CON group, 2206 DEGs were identified in the HF group and 1463 DEGs in the HF + ITA group relative to the HF group. KEGG enrichment analysis showed that these DEGs were significantly concentrated in the autophagy–lysosome pathway, with mTOR signaling annotated as an upstream regulator [57,58], suggesting that dietary ITA modulates transcriptional activity within the hepatic mTOR/autophagy/lysosome axis. To clarify these associations, we analyzed the mTOR/autophagy pathway and lysosomal biogenesis separately. HFD reduced the transcription of autophagy-initiation genes (*prkaa1*, *ulk2*, *map1lc3a*, *sqstm1*) while upregulating mTOR. It also markedly decreased the expression of lysosomal biogenesis-related genes (*ap3s2*, *igf2r*, *ap1g1*, *ap1m2*) and lysosomal hydrolase-encoding genes (*lgmn*, *ctso*, *ctsk*). These changes indicate that HFD may inhibit hepatic autophagy initiation via AMPK/mTOR signaling and impair lysosome formation and hydrolase production, thereby restricting autophagosome–lysosome fusion. Given the central role of autophagy in clearing hepatic lipid deposits [59,60], such disruption likely contributes to lipid accumulation under HFD conditions. qPCR validation confirmed that ITA counteracted these effects by upregulating *prkaa1*, *ulk2*, *map1lc3a*, *igf2r*, *ap3s2*, *lgmn*, and *ctso* while suppressing mTOR expression. These results indicate that ITA enhances the transcription of autophagy-initiation and lysosomal biogenesis-related genes through AMPK/mTOR-associated regulation, consistent with its observed capacity to reduce hepatic lipid accumulation. Previous work has shown that ITA alkylates Cys212 of TFEB, disrupts its interaction with mTOR and 14-3-3 proteins, and promotes TFEB nuclear translocation, where it activates lysosomal biogenesis programs [47]. Thus, by simultaneously promoting autophagy initiation and lysosomal formation, ITA may reinforce hepatic autophagic flux. This interpretation aligns with prior evidence that autophagy activation effectively alleviates hepatic lipotoxicity in fish [61,62]. Nonetheless, several experimental limitations must be acknowledged. The transcriptomic profiling was performed with a limited biological replicate (n = 3), and only a single ITA intratumoral administration dosage was examined, constraining the robustness and extrapolation of the findings. Furthermore, critical functional assays—such as quantification of protein phosphorylation states in the AMPK/mTOR pathway, autophagic flux markers, and lysosomal enzymatic activity assays—were not conducted. Future investigations should incorporate protein phosphorylation analyses, autophagic flux assessments, and lysosomal enzymatic function measurements to substantiate the mechanistic hypotheses presented.

In summary, ITA mitigates HFD-induced hepatic steatosis by activating autophagy via the AMPK/mTOR pathway and upregulating lysosome biogenesis genes, thereby enhancing lipophagy and decreasing liver lipid accumulation. Given the conserved AMPK/mTOR–lysosome pathway, ITA may offer comparable hepatoprotective effects in other aquaculture species, guiding future research on its development as a broad-spectrum lipid-lowering feed additive. ITA can be mass-produced via microbial fermentation at low cost, and its inclusion at 0.4% in feed is feasible and economical. Compared to other functional additives for FLD (plant extracts, selenium, organic acids, and L-carnitine), ITA exhibits superior availability, affordability, bio-compatibility, and a direct lipid-lowering mechanism, indicating strong commercialization potential. However, potential interactions between ITA and environmental stressors (temperature, hypoxia, crowding) under high-energy feeding conditions require further investigation.

## 5. Conclusions

The results of this study demonstrate that ITA effectively alleviates HFD-induced liver lipid accumulation in largemouth bass. The underlying mechanism primarily involves the AMPK/mTOR signaling pathway, through which ITA promotes the expression of autophagy initiation and lysosome biogenesis, thereby increasing autophagic flux and accelerating lipid degradation. Furthermore, ITA ameliorates HFD-induced growth suppression, improves fatty acid metabolism and physiological function in the liver, and enhances antioxidant capacity. In conclusion, as a safe and effective feed additive, itaconic acid shows potential application value in preventing and treating FLD in fish.

## Figures and Tables

**Table 1:** Ingredients and nutrient composition of 3 experimental diets (%, as air-dried basis).

Ingredients (%)	Con	HF	HF + ITA
Peru fish meal ^b^	50.00	50.00	50.00
Soybean protein concentrate ^b^	13.00	13.00	13.00
Soybean meal ^b^	10.00	10.00	10.00
Wheat flour	7.00	7.00	7.00
Soybean oil	3.00	7.00	7.00
Fish oil	3.00	7.00	7.00
Vitamin C	0.20	0.20	0.20
Sodium carboxymethyl cellulose	1.00	1.00	1.00
Vitamin premix ^c^	0.75	0.75	0.75
Mineral premix ^d^	0.75	0.75	0.75
Ca(H_2_PO_4_)_2_	1.50	1.50	1.50
Microcrystalline cellulose	9.80	1.80	1.40
ITA ^a^	0.00	0.00	0.40
Total	100.00	100.00	100.00
Nutrition analysis, % dry matter			
Crude protein	47.89	46.90	46.92
Crude lipid	10.56	17.88	18.03
Ash	12.38	13.98	16.60
Moisture	7.95	7.93	7.85

^a^ Purchased from Beijing Solarbio Science & Technology Co., Ltd. (Beijing, China), purity ≥ 98%. ^b^ Purchased from Tongwei Group Co., Ltd. (Chengdu, China), Peru fish meal: crude protein, 67.2%, crude lipid, 7.63%; soybean protein concentrate: crude protein, 65.16%; crude lipid, 0.52%; soybean meal: crude protein, 45.44%, crude lipid, 1.26%. ^c^ Vitamin premix (mg/kg): Vitamin B1, 17.80; Vitamin A, 16,000 IU; Vitamin B2, 48; Vitamin B6, 29.52; Vitamin B12, 0.24; Vitamin C, 800; Vitamin D3, 8000 IU; Vitamin E, 160; Vitamin K3, 14.72; Choline 1500 chloride; Folate, 6.40; Nicotinamide, 79.20; Inositol, 320; Calcium pantothenate, 73.60; Biotin, 0.64. ^d^ Mineral premix (mg/kg): iodine (Ca(IO_3_)_2_), 1.63; Manganese (MnSO_4_), 6.20; Copper (CuSO_4_), 2.00; Iron (FeSO_4_), 21.10; Selenium (Na_2_SeO_3_), 0.18; Cobalt (CoCl_2_), 0.24; Zinc (ZnSO_4_), 34.40.

**Table 2:** Juvenile largemouth bass primers for qRT-PCR analysis.

Target Gene	Primer Sequence (5′–3′)	Size (bp)	Accession
*maplc3a*	F: TTTGCTGACCGGTGCAAAGA	173	XM_038697699.1
	R: CGACGCCTGATGATCTTGAC		
*ulk2*	F: CGATGTCCAGGAAACACCCA	213	XM_038694231.1
	R: CCCGCATATGACAGCAGGAT		
*prkaa1*	F: GAATTAGCAAGCCCCACCCT	197	XM_038734014.1
	R: CTGTTTCATTGCACGGCACA		
*lgmn*	F: AAAGTACTGAAGAGCGGCCC	183	XM_038728731.1
	R: AGACTCGCATGCCTCAATGT		
*ap3s2*	F: GATCGTAGCCCAGGTCGAAG	109	XM_038700750.1
	R: CTCGGGCAGGTTCATGTTCT		
*ctso*	F: ACCAACAAGCATGTGGAAGT	239	XM_038699159.1
	R: GCAGATTCCTGTCTTGGCCT		
*mTOR*	F: ATCGTGACGACAGGGTCCAT	266	XM_038723321.1
	R: GGTGAGGAAGCCTTGAGGTG		
*igf2r*	F: ATGACTGTCGGGTTCGTGAC	206	XM_038727258.1
	R: TGCCATTCCTGCGATCTTGT		
*β-actin*	F: CTCTGCATACATGCCTACAC	117	XM_038695351.1
	R: GTAGAGTTTCTCCCCATCAGG		
*EF1α*	F: TGCTGCTGGTGTTGGTGAGTT	147	XM_038724777.1
	R: TTCTGGCTGTAAGGGGGCTC		

Note: *maplc3a*: microtubule-associated protein 1 light chain 3 alpha; *ulk2*: unc-51 like autophagy activating kinase 2; *prkaa1*: protein kinase, AMP-activated, alpha 1 catalytic subunit; *lgmn*: legumain; *ap3s2*: adaptor related protein complex 3 subunit sigma 2; *ctso*: cathepsin O; *mTOR*: serine/threonine-protein kinase mTOR; *igf2r*: insulin-like growth factor 2 receptor. The capital “F” in the table denotes the forward primer, while the capital “R” denotes the reverse primer.

**Table 3:** Effect of ITA supplementation on growth performance of juvenile largemouth bass fed a high-fat diet.

Parameters	CON	HF	HF + ITA
IBW g/per fish	2.25 ± 0.01	2.25 ± 0.00	2.25 ± 0.01
FBW g/per fish	17.96 ± 0.57 ^c^	13.84 ± 0.32 ^a^	15.51 ± 0.24 ^b^
WG g/per fish	15.71 ± 0.57 ^c^	11.59 ± 0.32 ^a^	13.26 ± 0.24 ^b^
WGR%	698.48 ± 26.33 ^c^	514.36 ± 13.33 ^a^	588.38 ± 11.08 ^b^
SGR%/d	2.87 ± 0.03 ^c^	2.61 ± 0.02 ^a^	2.73 ± 0.02 ^b^
FI g/per fish	17.32 ± 0.81 ^b^	16.52 ± 0.20 ^ab^	15.49 ± 0.12 ^a^
FCR	1.15 ± 0.03 ^a^	1.51 ± 0.04 ^b^	1.27 ± 0.01 ^a^

Note: ITA = Itaconic acid; FBW = final mean body weight; IBW = initial mean body weight; FCR = feed conversion ratio; FI = food intake; SGR = specific growth rate; WGR = weight gain rate; WG = weight gain. CON: The feed contains 10% lipid without the addition of ITA. HF: The feed contains 18% lipid without the addition of ITA. HF + ITA: The feed contains 18% lipid with the addition of 0.4% ITA. Data are presented as mean ± SEM (n = 3). Statistical differences are represented by different letters (*p* < 0.05).

**Table 4:** Effect of ITA supplementation on liver lipid metabolism-related parameters of juvenile largemouth bass fed a high-fat diet.

Parameters	CON	HF	HF + ITA
BL cm	11.30 ± 0.15 ^c^	9.72 ± 0.04 ^a^	10.67 ± 0.04 ^b^
LW g	0.15 ± 0.00 ^a^	0.19 ± 0.00 ^c^	0.17 ± 0.00 ^b^
HSI (%)	0.83 ± 0.01 ^a^	1.37 ± 0.02 ^c^	1.07 ± 0.03 ^b^
CF (%)	1.24 ± 0.01 ^a^	1.51 ± 0.02 ^b^	1.28 ± 0.03 ^a^
AFW g	0.11 ± 0.00 ^a^	0.22 ± 0.02 ^b^	0.14 ± 0.00 ^a^
ABR	0.63 ± 0.02 ^a^	1.59 ± 0.09 ^c^	0.90 ± 0.01 ^b^

Note: ITA = Itaconic acid; ; BL = body length; LW = liver weight; HSI = hepatic steatosis index; CF = condition factor; AFW = abdominal fat weight; ABR = ratio of abdominal fat weight to body weight. CON: The feed contains 10% lipid without the addition of ITA. HF: The feed contains 18% lipid without the addition of ITA. HF + ITA: The feed contains 18% lipid with the addition of 0.4% ITA. Data are presented as mean ± SEM (n = 3). Statistical differences are represented by different letters (*p* < 0.05).

**Table 5:** Effect of ITA supplementation on liver biochemical parameters of juvenile largemouth bass fed a high-fat diet.

Parameters	CON	HF	HF + ITA
TP g/L	10.77 ± 0.78	9.28 ± 1.26	10.35 ± 0.35
TG mmol/g prot	0.25 ± 0.02 ^a^	0.49 ± 0.04 ^b^	0.30 ± 0.02 ^a^
TCHO mmol/g prot	0.02 ± 0.00 ^a^	0.04 ± 0.00 ^b^	0.03 ± 0.00 ^a^
LDL μmol/g prot	0.06 ± 0.01	0.03 ± 0.00	0.06 ± 0.01
HDL μmol/mg prot	17.55 ± 1.32 ^a^	40.59 ± 1.85 ^b^	23.03 ± 2.43 ^a^
ALT U/g prot	64.64 ± 3.20 ^a^	103.90 ± 2.03 ^b^	62.31 ± 1.25 ^a^
AST U/g prot	83.57 ± 1.28 ^a^	108.24 ± 1.86 ^b^	89.19 ± 5.69 ^a^

Note: ITA = Itaconic acid; TP = total protein content; TG = total triglycerides; TCHO = total cholesterol; LDL = low-density lipoprotein; HDL = high-density lipoprotein; ALT = alanine aminotransferase; AST = aspartate aminotransferase. CON: The feed contains 10% lipid without the addition of ITA. HF: The feed contains 18% lipid without the addition of ITA. HF + ITA: The feed contains 18% lipid with the addition of 0.4% ITA. Data are presented as mean ± SEM (n = 3). Statistical differences are represented by different letters (*p* < 0.05).

**Table 6:** Effect of ITA supplementation on serum antioxidant indexes of juvenile largemouth bass fed a high-fat diet.

Parameters	CON	HF	HF + ITA
H_2_O_2_ mmol/L	71.21 ± 4.19 ^a^	93.63 ± 6.40 ^b^	56.97 ± 3.90 ^a^
T-AOC mM	0.35 ± 0.02 ^c^	0.19 ± 0.00 ^a^	0.25 ± 0.01 ^b^
SOD U/L	3.21 ± 0.13 ^b^	2.34 ± 0.15 ^a^	3.16 ± 0.04 ^b^
CAT U/mL	6.14 ± 0.87 ^b^	3.23 ± 0.42 ^a^	5.58 ± 0.91 ^ab^
MDA nmol/L	8.83 ± 2.12 ^a^	25.41 ± 1.74 ^b^	9.19 ± 1.43 ^a^
GPX U/L	339.10 ± 8.36 ^b^	282.99 ± 3.10 ^a^	342.69 ± 11.39 ^b^

Note: ITA = Itaconic acid; H_2_O_2_ = hydrogen peroxide; T-AOC = total antioxidant capacity; SOD = superoxide dismutase; CAT = catalase; MDA = malondialdehyde; GPX = glutathione peroxidase. CON: The feed contains 10% lipid without the addition of ITA. HF: The feed contains 18% lipid without the addition of ITA. HF + ITA: The feed contains 18% lipid with the addition of 0.4% ITA. Data are presented as mean ± SEM (n = 3). Statistical differences are represented by different letters (*p* < 0.05).

**Table 7:** Effect of ITA supplementation on whole-body proximate composition of juvenile largemouth bass fed a high-fat diet.

Parameters	CON	HF	HF + ITA
Moisture	71.77 ± 0.31	70.20 ± 1.32	70.87 ± 1.19
Crude protein	48.52 ± 0.28	48.38 ± 0.55	47.24 ± 0.33
Crude lipid	31.52 ± 0.83 ^a^	37.63 ± 0.59 ^b^	33.50 ± 0.55 ^a^
Ash	16.32 ± 0.38	14.61 ± 0.49	14.00 ± 0.71

Note: ITA = Itaconic acid. CON: The feed contains 10% lipid without the addition of ITA. HF: The feed contains 18% lipid without the addition of ITA. HF + ITA: The feed contains 18% lipid with the addition of 0.4% ITA. Data are presented as mean ± SEM (n = 3). Statistical differences are represented by different letters (*p* < 0.05).

## Data Availability

The original contributions presented in this study are included in the article and Supplementary Materials. Further inquiries can be directed to the corresponding author.

## Supplementary Materials

The following supporting information can be downloaded at: https://www.mdpi.com/article/10.3390/antiox15010006/s1.

## Abbreviations

The following abbreviations are used in this manuscript:

ITA	Itaconic Acid
HFD	High-Fat Diet
CON	Control Diet
HF + ITA	High-Fat Diet with ITA supplementation
FBW	Final Body Weight
IBW	Initial Body Weight
WG	Weight Gain
WGR	Weight Gain Rate
SGR	Specific Growth Rate
FI	Feed Intake
FCR	Feed Conversion Ratio
BL	Body Length
LW	Liver Weight
HSI	Hepatosomatic Index
CF	Condition Factor
AFW	Abdominal Fat Weight
ABR	Abdominal Fat to Body Weight Ratio
TP	Total Protein
TG	Triglycerides
TCHO	Total Cholesterol
LDL	Low-Density Lipoprotein
HDL	High-Density Lipoprotein
ALT	Alanine Aminotransferase
AST	Aspartate Aminotransferase
H_2_O_2_	Hydrogen Peroxide
T-AOC	Total Antioxidant Capacity
SOD	Superoxide Dismutase
CAT	Catalase
MDA	Malondialdehyde
GPX	Glutathione Peroxidase
q-PCR	Quantitative Real-Time PCR
DEGs	Differentially Expressed Genes
KEGG	Kyoto Encyclopedia of Genes and Genomes
PCR	Polymerase Chain Reaction
cDNA	Complementary DNA
RNA	Ribonucleic Acid
AOAC	Association of Official Agricultural Chemists
SEM	Standard Error of the Mean
*AMPK*	AMP-activated Protein Kinase
*mTOR*	Mechanistic Target of Rapamycin
*prkaa1*	Protein Kinase AMP-Activated Catalytic Subunit Alpha 1
*ulk2*	Unc-51 Like Autophagy Activating Kinase 2
*map1lc3*	Microtubule Associated Protein 1 Light Chain 3
*sqstm1*	Sequestosome 1
*igf2r*	Insulin Like Growth Factor 2 Receptor
*ap3s2*	Adaptor Related Protein Complex 3 Subunit Sigma 2
*lgmn*	Legumain
*ctso*	Cathepsin O
*ctsk*	Cathepsin K
*ap1g1*	Adaptor Related Protein Complex 1 Subunit Gamma 1
*ap1m2*	Adaptor Related Protein Complex 1 Subunit mu 2
