# Supplementary material for: Itaconic Acid Activates Lysosomal Biogenesis and Autophagy Flux and Mitigates High-Fat Diet-Induced Liver Lipid Accumulation in Largemouth Bass (Micropterus salmoides)
Source: Antioxidants (Basel). 2025 Dec 20;15(1):6. doi: 10.3390/antiox15010006 (PMC12838206; doi:10.3390/antiox15010006)
Supplement: Supplementary file 1 [file antioxidants-15-00006-s001.zip › Supplemental data.pdf]

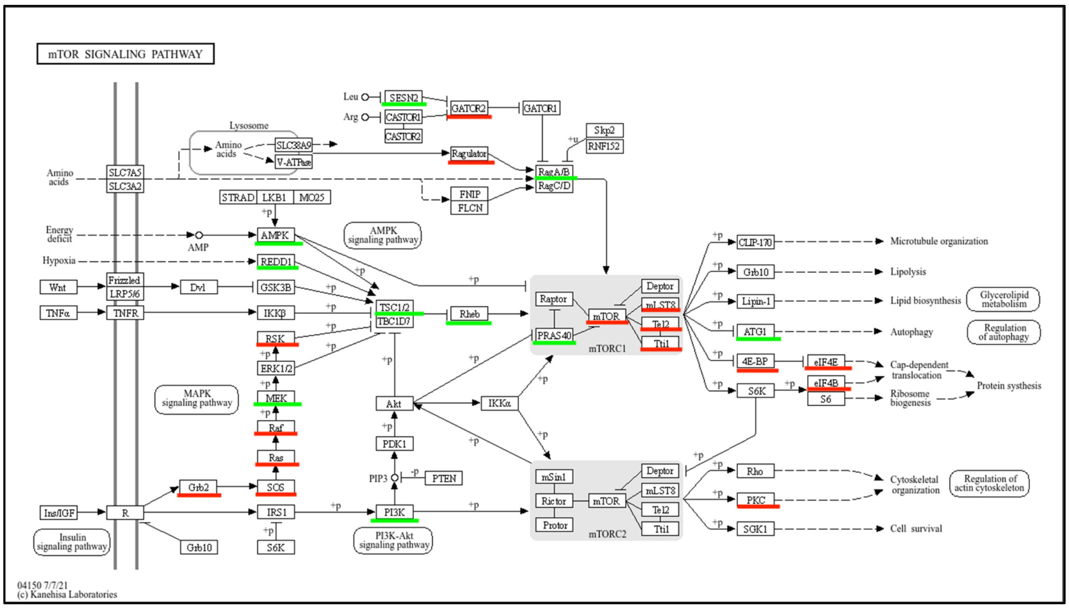

(A)

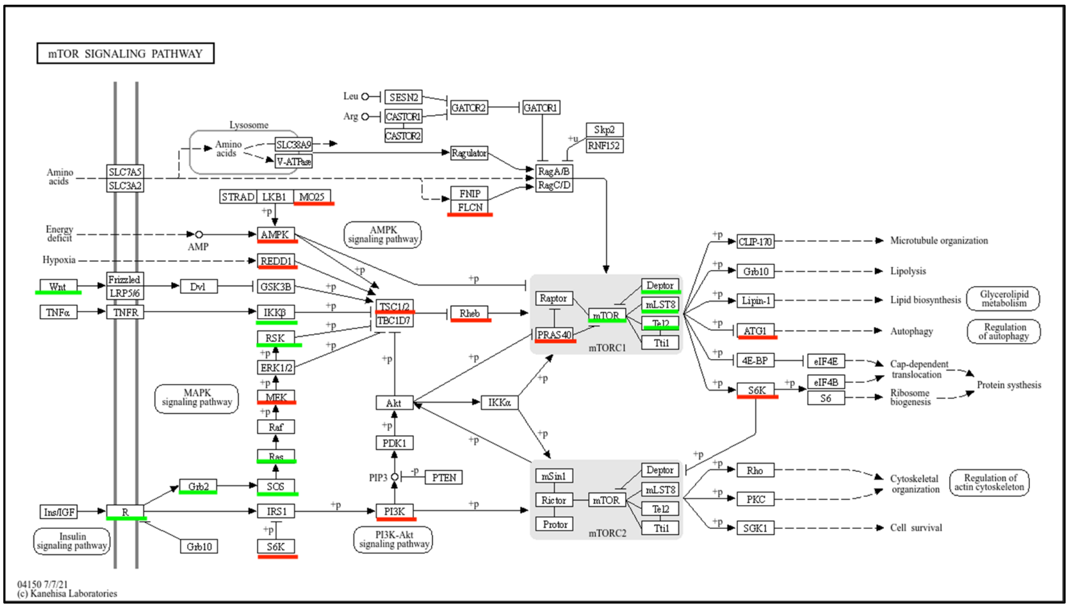

(B)

Supplemental Figure S1: Effects of dietary supplementation with 0.4% ITA on liver mTOR signalling pathway of largemouth bass fed a high-fat diet (n=3). Mapping and visualization of gene expression profiles on KEGG pathways between (A) CON vs HF and (B) HF vs HF+ITA groups. The red underlining indicates genes that are markedly upregulated, while the green underlining signifies genes that are markedly downregulated.

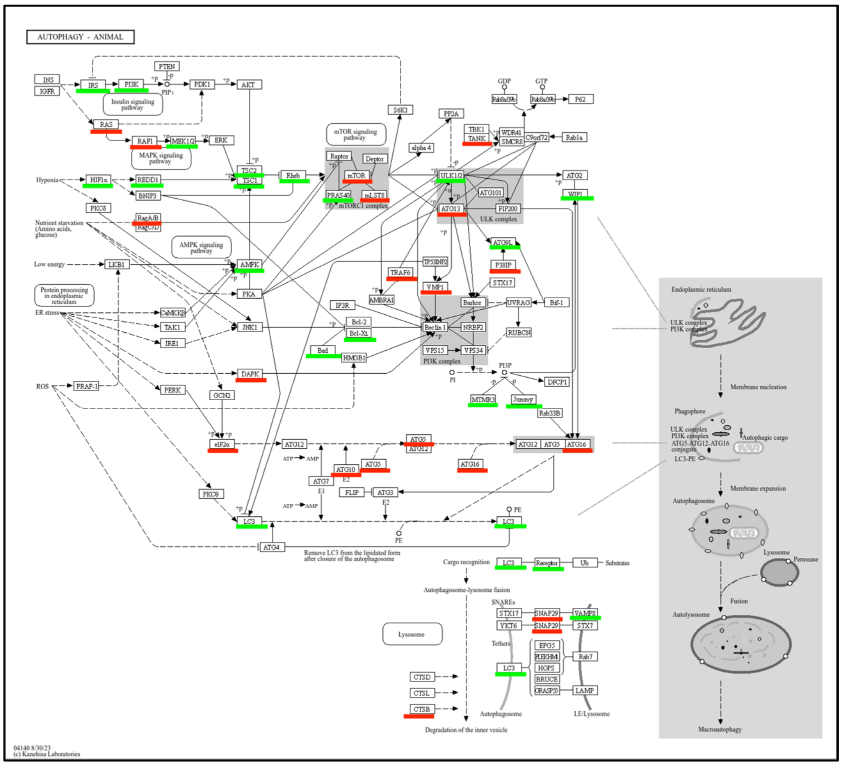

(A)

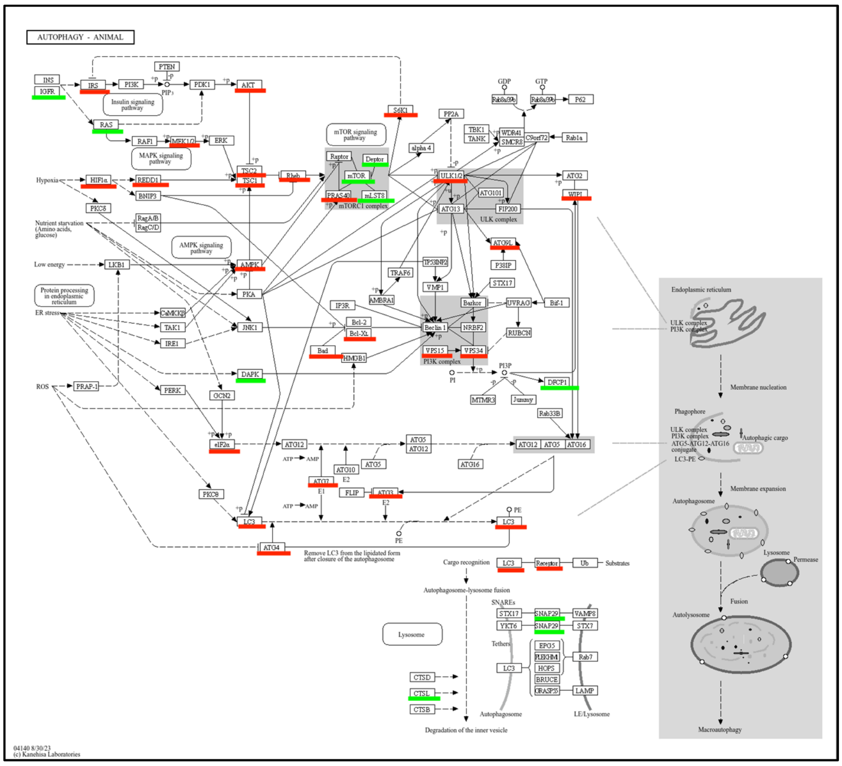

(B)

Supplemental Figure S2: Effects of dietary supplementation with 0.4% ITA on liver Autophagy-animal of large-mouth bass fed a high-fat diet (n=3). Mapping and visualization of gene expression profiles on KEGG pathways between (A) CON vs HF and (B) HF vs HF+ITA. The red underlining indicates genes that are markedly up-regulated, while the green underlining signifies genes that are markedly downregulated.

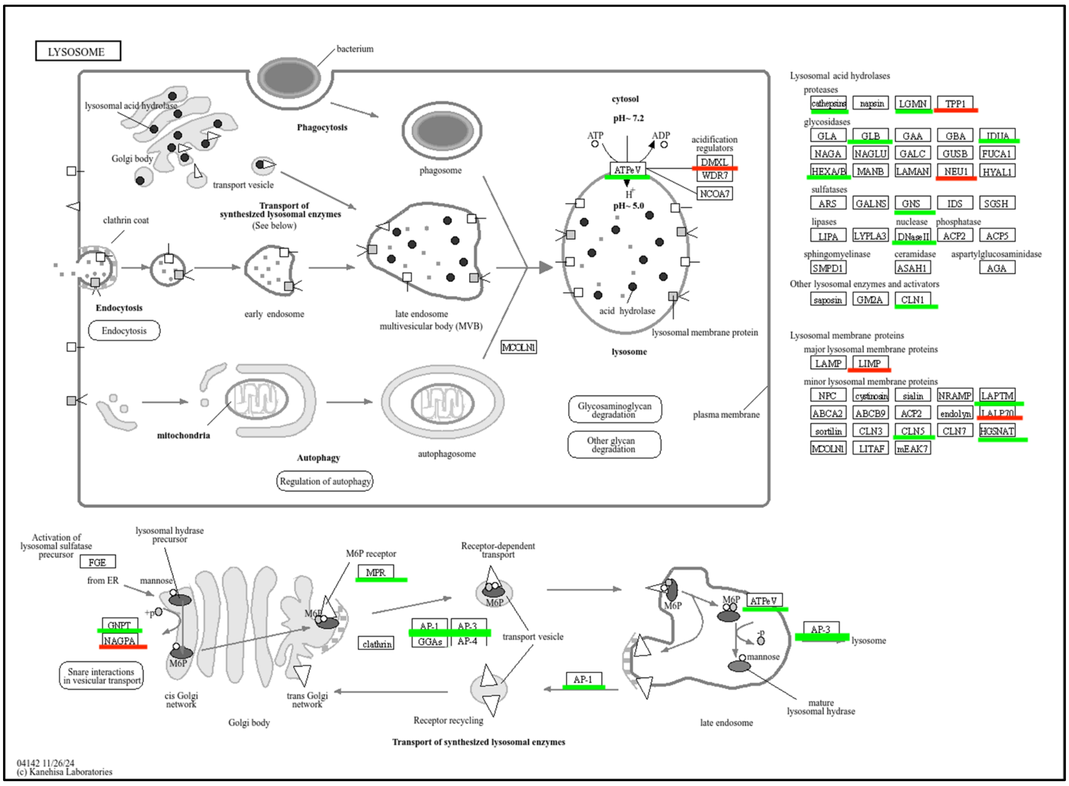

(A)

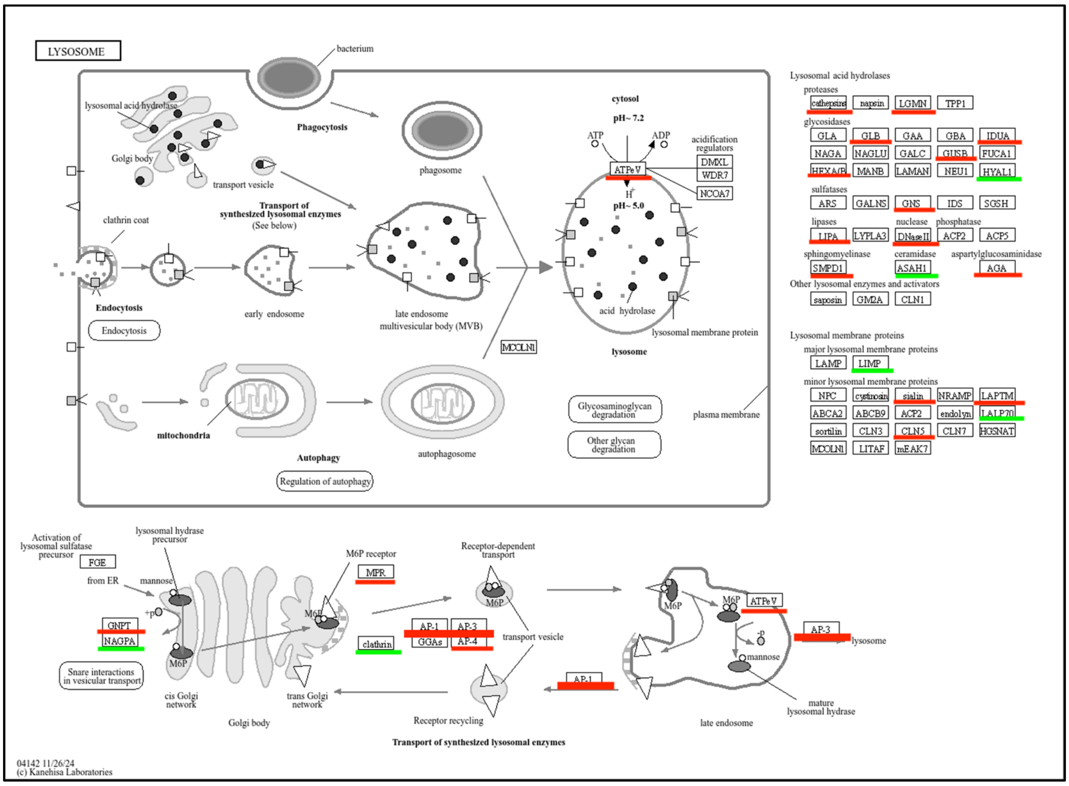

(B)

Supplemental Figure S3: Effects of dietary supplementation with 0.4% ITA on liver Lysosome of largemouth bass fed a high-fat diet (n=3). Mapping and visualization of gene expression profiles on KEGG pathways between (A) CON vs HF and (B) HF vs HF+ITA. The red underlining indicates genes that are markedly upregulated, while the green underlining signifies genes that are markedly downregulated.
